# Effectiveness of a team intervention in reducing modifiable cardiovascular disease risk in HIV-infected subjects on antiretroviral therapy

**DOI:** 10.7448/IAS.17.4.19546

**Published:** 2014-11-02

**Authors:** Mark Bloch, Avindra Jayewardene, Trina Vincent, Natalie Linton, Dick Quan, Andrew Gowers

**Affiliations:** Clinical Research Department, Holdsworth House Medical Practice, Darlinghurst, Australia

## Abstract

**Introduction:**

The increasing age, higher modifiable and inherent cardiovascular disease (CVD) risk of HIV-infected patients [1] necessitates improved approaches to reducing co-morbidities. We aimed to assess the effectiveness of a team intervention in reducing modifiable CVD risk.

**Materials and Methods:**

HIV-infected patients ≥50 years attending a large HIV caseload primary-care practice, who were virologically suppressed on antiretroviral therapy (ART), with moderate or severe 10-year CVD Framingham risk (≥10%) were recruited for this prospective case-control study. Intervention participants were provided a team approach to care, which involved treatment by study doctors for lipid, hypertension and ART management, and monthly review by a team of research nurses and dieticians for smoking cessation, exercise and dietary advice over 12 months. Controls were matched on age and smoking status, and were given standard of care (SOC) by non-study doctors. Outcomes included CVD risk factors, body composition and CVD risk assessment, including Framingham 10-yr risk [2] and D:A:D 5-year estimated risk of coronary heart disease (CHD) [3]. Repeated measures analysis of variance was used to examine pre- and post-intervention differences, with p-values used to assess time and main effects of approach to care (Intervention, SOC).

**Results:**

A total of 33 patients completed the intervention, with 33 controls (58.0±6.8 and 59.1±6.9 years, respectively). Smoking cessation occurred in 25% cases versus nil controls. There was a significant change in CVD risk between intervention and control groups, in both Framingham scores (time and group×time interaction) and D:A:D scores (group×time interaction only) (Table 1). There was also a significant difference in change in total cholesterol over the study period (time and group×time interaction). Body composition was only measured in intervention patients, with a significant loss in % body fat observed in pre- and post-intervention.

**Conclusions:**

Team intervention was significantly more effective than standard of care in reducing CVD risk in HIV-infected patients on ART. A team approach to care may be an important component of reducing CVD risk in this population.

**Table 1:** Descriptive characteristics of participants at baseline and the change over the 12-month study

Variable	Intervention Baseline	Control Baseline	Intervention Δ from baseline	Control Δ from baseline	P (t effect)	P (g×t interaction)
Framingham 10-year risk (%)	19.6±6.6	21.2±8.9	−3.4±5.7	−0.3±5.9	0.013	0.033
D:A:D 5-year risk (%)	6.4±5.9	5.4±4.9	−1.4±3.1	0.6±3.3	NS	0.017
Total cholesterol (mmol/L)	5.2±1.0	4.9±1.1	−0.6±0.9	0.0±0.9	0.011	0.013
High-density lipoprotein (mmol/L)	1.2±0.3	1.2±0.3	0.5±0.2	0.1±0.3	NS	NS
Systolic blood pressure (mmHg)	130±13	132±12	−6.2±17.1	−0.6±12.4	NS	NS
% Body fat	22.3±4.2	–	−1.0±1.9	–	0.009	-

Descriptive characteristics of participants at baseline and the change over the 12-month study programme. Mean±SD; NS, not significant.
